# T Cell/Histiocyte-Rich Large B Cell Lymphoma of the Thymus: A Diagnostic Pitfall

**DOI:** 10.1155/2016/2942594

**Published:** 2016-01-20

**Authors:** Jie Xu, Xiaojun Wu, Vishnu Reddy

**Affiliations:** ^1^Department of Pathology, University of Alabama at Birmingham, Birmingham, AL 35244, USA; ^2^Department of Hematopathology, MD Anderson Cancer Center, Houston, TX 77030, USA; ^3^Department of Pathology, George Washington University Hospital, Washington, DC 20037, USA

## Abstract

T cell/histiocyte-rich large B cell lymphoma (THRLBCL) is predominantly a nodal disease, with extranodal involvement, such as bone marrow, spleen, and liver. However, primary THRLBCL has never been reported in the thymus in the English literature. Here we report a case of THRLBCL presenting as mediastinal mass and lymphadenopathy. Based on the frozen section diagnosis of “thymoma,” a 12 cm mass was excised. A year later she developed multiple enlarged lymph nodes and pulmonary nodules. Consultant review of the excised mediastinal mass showed scattered large atypical cells that were CD20+ and PAX-5+ and negative for pan-cytokeratin, AE1, and AE3, compatible with THRLBCL and excluding thymoma. The excised lymph nodes were replaced by diffuse infiltrate of small CD3+ lymphocytes and histiocytes with intermingled large CD20+ B lymphoma cells scattered throughout the section. A diagnosis of THRLBCL was made in lymph node, similar to previous thymic lesion. A clonal rearrangement of immunoglobulin heavy chain (IGH) gene was detected, further supporting the diagnosis. This is the first reported case of THRLBCL in thymus. Correct recognition of this entity is critical, because of the difference in therapeutic impact on these patients.

## 1. Introduction

The thymus is the primary site of T cell development; however, both T and B cell lymphomas can arise from thymus. Hodgkin's lymphoma, primary mediastinal (thymic) large B cell lymphoma [PMBL, a subtype of diffuse large B cell lymphoma (DLBCL)], and lymphoblastic lymphoma are the most common thymic lymphoid neoplasms. T cell/histiocyte-rich large B cell lymphoma (THRLBCL) is a morphologic variant of DLBCL characterized by fewer than 10% large neoplastic cells amid a prominent inflammatory infiltrate, the majority of which are small polyclonal T cells with or without the presence of histiocytes, and it has never been reported in the thymus in the English literature to date. THRLBCL is predominantly a nodal disease, but extranodal sites, such as bone marrow, liver, and spleen, can be involved [1]. Herein we report the first case of THRLBCL arising from thymus. The present patient also had a history of Sjögren's syndrome (SS), a chronic autoimmune epithelitis characterized by lymphocytic infiltration of exocrine glands together with polyclonal B cell activation [2, 3]. Patients with SS have increased risk for lymphoma and the prevalence of non-Hodgkin lymphoma (NHL) in SS is about 5% [4]. Various subtypes of NHL have been described in SS, with marginal zone lymphoma of mucosa-associated lymphoid tissue (MALT lymphoma) being the most common one. However, THRLBCL is extremely rare in SS patients with only one case reported in the parotid gland so far [5].

## 2. Case Report

**Figure 1:**
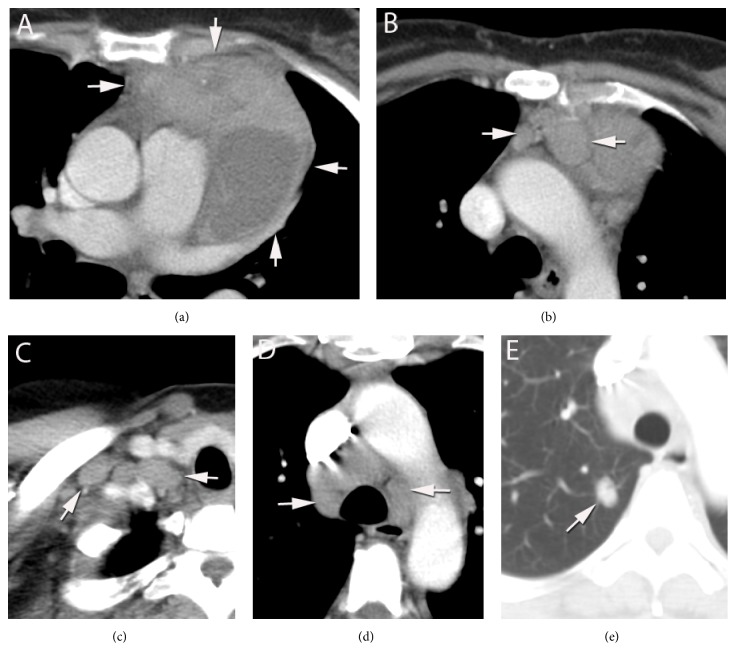
Chest computed tomography (CT) scan. Before resection of the mediastinal mass, chest CT scan showed a 12 cm anterior mediastinal mass (a) and lymph nodes (b). A year after the surgery, CT scan revealed multiple enlarged lymph nodes, such as cervical lymph nodes (c), paratracheal lymph node (d), and pulmonary nodules (e). Arrows indicated the lesions.

The patient was a 52-year-old African American woman who had a long-standing history (>10 years) of SS and had been treated with corticosteroid. She was admitted to a regional medical center for a sudden onset of chest pain. CT scan showed a large anterior mediastinal mass and several enlarged mediastinal lymph nodes (Figures 1(a) and 1(b)); liver and spleen were unremarkable. Her HIV serology was negative. During the surgery, a mass was identified extending from the right side of midline all the way over into the left chest into the hilum of the left lung, adhering to the pericardium as well as to the pleural surface on the left side and involving the phrenic nerve. The intraoperative pathological diagnosis of the mass was reported as “thymoma” and the mass and mediastinal lymph nodes were then excised and submitted for pathology. On gross examination, a large (12 × 9.5 × 7 cm, 237 grams), irregular, pink to dark red mass had pink-tan, lobular appearing cut surface separated by bands of yellow-tan tissue. Areas of ischemia or necrosis were present. Multiple lymph nodes (0.5–1.3 cm) were noted at one end of the mass. The permanent sections of the mass and lymph nodes were reported as “thymoma, lymphocyte predominant type; benign reactive lymph nodes.” The patient's postoperative course was uneventful. However, a year later, a follow-up CT showed multiple enlarged lymph nodes throughout the chest and upper abdomen, multiple new noncalcified pulmonary nodules, and bilateral pulmonary dense consolidation (Figures 1(c)–1(e)). The patient also reported night sweats, low grade fever, flu-like symptoms, green sputum production, 14 lb weight loss, and some shortness of breath with exertion. Biopsies of multiple lymph nodes (right cervical and bilateral lower paratracheal) were performed. Pathology material from previous mediastinal mass resection and current lymph node biopsy were sent to our medical center for review and consultation.

**Figure 2:**
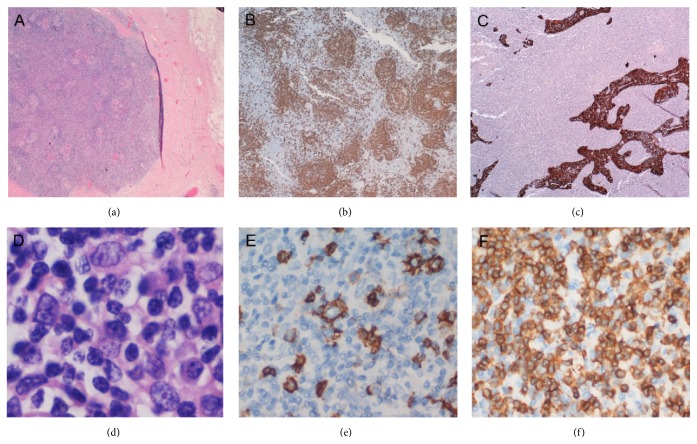
Mediastinal mass, thymic THRLBCL. (a) Thymic tissue rimmed by collagenous fibrous capsule (H&E, 2x). (b) Nodular areas of B cells (CD20, 4x). (c) Distorted and compressed epithelial network (AE1 and AE3, 2x). (d) Large lymphoid cells were scattered singly among small lymphocytes (H&E, 100x). (e) The dispersed large B cell lymphoma cells and some small B lymphocytes (CD20, 40x). (f) Numerous T lymphocytes in the background (CD3, 40x).

Consultative review of the mediastinal mass sections showed thymic tissue rimmed by collagenous fibrous capsule (Figure 2(a)). Hyperplastic lymphoid infiltrate was present consisting of diffuse CD3+ T cells (predominantly), nodular areas of CD20+ B cells, and distinct, well formed germinal centers (Figure 2(b)). Preserved follicular dendritic network (CD21+) was present in the germinal centers. These features are consistent with thymic lymphoid follicular hyperplasia. This diffuse lymphoid hyperplasia resulted in an indistinct border between thymic cortex and medulla, a distorted and compressed epithelial network (highlighted by immunostains for AE1 and AE3; Figure 2(c)), and markedly decreased number of Hassall's corpuscles. Scattered large atypical cells were present in the interfollicular area.

Furthermore, focally effaced areas contained large atypical lymphoid cells in a background of small lymphoid cells (Figures 2(d) and 2(f)). The large atypical cells had irregular nuclei, vesicular chromatin, and one or more nucleoli and were CD20+ (Figure 2(e)), PAX5+, and CD30+ (subsets only) but were negative for CD15 and EBER (in situ hybridization). A proliferation fraction of 15–30% by Ki-67 was identified in the areas with large atypical B lymphocytes. Patchy areas of necrosis were also present. No prominent histiocytic proliferation was identified in the thymic mass. Epithelial proliferation, as seen in thymoma, was absent, which was confirmed by several negative immunostains for different keratin markers, including pan-cytokeratin, AE1, and AE3. The histomorphologic features and the immunophenotype resulted in a diagnosis of thymic THRLBCL.

**Figure 3:**
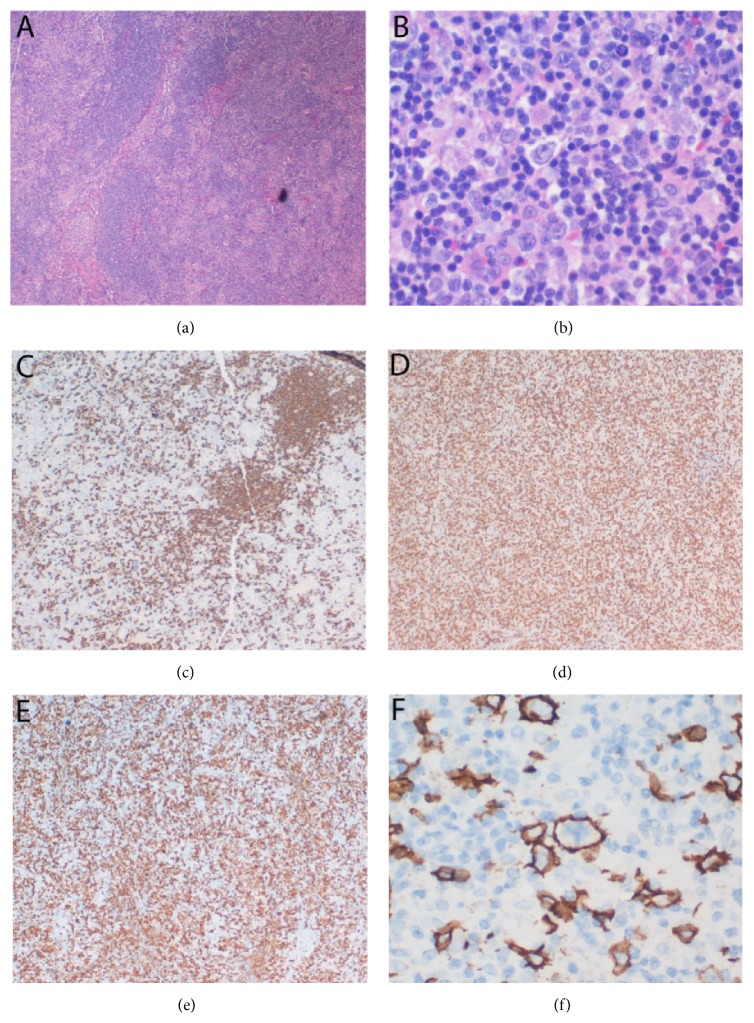
THRLBCL in the right cervical lymph node. (a) The lymph node structure was almost entirely effaced by diffuse small lymphocytes with some residual follicles present (H&E, 4x). (b) Large atypical cells were surrounded by small lymphocytes and histiocytes (H&E, 40x). (c) CD20 immunostain showed the residual follicles (4x). (d) The diffuse small lymphocytes were CD3+ T lymphocytes (4x). (e) Many histiocytes were stained by CD163 (4x). (f) Immunohistochemistry for CD20 highlighted large B lymphoma cells (40x).

**Figure 4:**
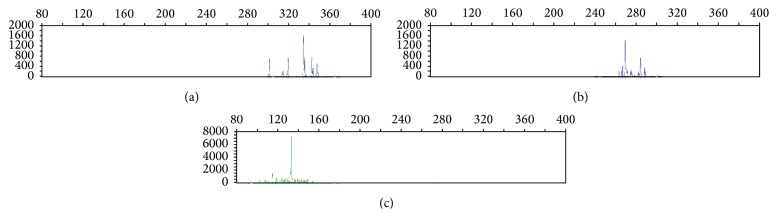
B cell clonality detection by PCR (IGH-PCR). A clonal IGH gene rearrangement was identified in framework 3 region (c), but not in framework 1 (a) or 2 (b) region.

In addition, the mediastinal lymph node from previous mediastinal mass resection and the bilateral lower paratracheal and right cervical lymph nodes from recent biopsy showed similar morphology as follows: mostly or entirely effaced lymph node architecture by predominantly small CD3+ lymphocytes, with residual nodular CD20+ B lymphoid areas and CD21+ germinal centers present in some sections (Figures 3(a), 3(c), and 3(d)). Interspersed large atypical lymphocytes (Figure 3(b)) were present in the background of T lymphocytic (CD3+) and histiocytic (CD68+, CD163+) proliferation (Figures 3(d) and 3(e)). The large atypical lymphoid cells were CD20+, PAX-5+, MUM-1+, and CD30+ (subsets only) but showed no reactivity to CD15, S-100, or EBER (in situ hybridization) (Figure 3(f)). Immunostain for Ki-67 revealed a proliferation fraction of 30–40% among large atypical lymphoid cells. No epithelial cells were identified by immunostains for pan-cytokeratin, AE1, and AE3. No acid fast bacilli or fungal organisms were identified by ZN and GMS stains. The histopathological changes of the lymph nodes were consistent with THRLBCL. Polymerase chain reaction (PCR) detected a clonal rearrangement of immunoglobulin heavy (IGH) chain (Figure 4(c)), further supporting the B cell neoplastic process.

## 3. Discussion

Malignant lymphoma is the second most common primary anterior mediastinal tumors in adults [6]. The thymus, located in the anterior mediastinum, is the primary anatomic site of T cell development. Therefore, it is not surprising that many lymphoid malignant neoplasms arising within the thymus are T cell neoplasms, mostly T cell lymphoblastic lymphoma. However, malignant neoplasms of B cell lineage, such as Hodgkin's lymphoma and PMBL, are not uncommon in thymus [7]. Several studies have documented the existence of a minor population of B cells in the thymus of both humans and mice [8]. These cells are located in the medulla, primarily around Hassall corpuscles, constituting approximately one-third of all medullary cells and may play a role in thymic negative selection. Hodgkin's lymphoma, PMBL, and lymphoblastic lymphoma are the most common thymic lymphoid neoplasms.

In the present patient, the large atypical cell scattered in the background of small lymphocytes led to a list of histological differential diagnosis: THRLBCL, Hodgkin's lymphoma, and thymoma of lymphocytes predominant type. THRLBCL is a variant of DLBCL and accounts for <10% of all DLBCL. THRLBCL is associated with a prominent component of reactive T cells, which may be related to interleukin-4 production by the lymphoma cells [9]. Previous studies show evidence that IL-4 may play a role in the proliferation of T cells and/or the suppressor of B cell growth (although it is still subject to investigation). The presence of IL-4 in THRLBCLs but not in the other DLBCLs and reactive lymph nodes suggests that IL-4 may be a major factor involved in the pathology of THRLBCLs and it is an important factor for the differential diagnosis of this entity. THRLBCL mainly affects middle-aged man and is predominantly a nodal disease, but extranodal sites, such as bone marrow, liver, and spleen, can be involved [1]. However, no cases of THRLBCL have been reported in the thymus. THRLBCL usually presents with higher stage of disease and more commonly involves bone marrow and spleen compared to conventional DLBCL. Histologically, large lymphoid cells are scattered singly among small lymphocytes (reactive CD3+CD5+CD8+ T cells). The relative low number of neoplastic cells may be partially explained by tumor cell apoptosis mediated by cytotoxic CD8+ T cells [10]. The large cells are pleomorphic with irregular nuclei, expressing pan-B markers and BCL6, and are negative for CD15, CD30, CD5, CD10, and EBV. BCL2 and EMA can be variable positive [1].

The histology of THRLBCL can mimic Hodgkin's lymphoma, such as lymphocyte-rich type of classical Hodgkin's lymphoma (LRCHL) and nodular lymphocyte predominant Hodgkin's lymphoma (NLPHL). In LRCHL, the Reed-Sternberg cells are usually positive for CD15, CD30, and EBV but negative for pan-B markers or heterogenous staining if positive. The present case was positive for B markers (CD20 and PAX-5) but negative for CD15 and EBV, though CD30 was weakly and focally positive, which mitigated against the diagnosis of LRCHL. NLPHL usually shows nodules containing numerous small lymphocytes and a variable number of variant Hodgkin's cells (popcorn cells) which are usually positive for CD45 and CD20 but negative for CD15 and CD30. However, the surrounding small lymphocytes in NLPHL are usually a mixture of B cells (predominately) and T cells (though rich T cells can be seen in late stage of NLPHL), and CD57+ or PD-1+ T cells rosette around the large neoplastic B cells [11]. In the present case, the small lymphocytes in the background were predominantly CD3+ T cells with no T cells rosette around the large neoplastic cells, making NLPHL unlikely. Another important differential diagnosis for the present case is thymoma of lymphocyte predominant type. Thymoma is characterized by neoplastic epithelial proliferation. Even if lymphocytes can be rich in thymoma, type B1, epithelial cell proliferation should be found in the lymphoid component. By immunohistochemistry, the present case did not show any epithelial proliferation in the thymus, ruling out thymoma. Furthermore, the surgical findings of the mediastinal mass, such as adhesion to the surrounding tissues and invasion to the phrenic nerve, also highly suggested a malignant and aggressive process, though thymoma at higher clinical stage can be locally aggressive. Unfortunately, no further suspicion for malignancy was raised until the patient developed a widely spread disease and B symptoms in a year after initial surgery.

It is also noted in the present case that few histiocytes were identified in the thymic lymphoma, whereas abundant histiocytes were present in the nodal lymphoma. It is not clear why there was such significant difference in the amount of histiocytes between the patient's thymic mass and involved lymph nodes. Although studies have suggested that THRLBCL cases with less (≤70%) or more (>70%) T cell infiltration significantly differ in clinical presentation or outcome [12], it is unknown if the amount of histiocytes is relevant to the disease progression and prognosis.

The present patient's development of lymphoma might be related to her long-standing history of SS. SS is a chronic autoimmune epithelitis characterized by lymphocytic infiltration of exocrine glands together with polyclonal B cell activation [2, 3]. The clinical spectrum of SS extends from autoimmune exocrinopathy to a systemic disease affecting the musculoskeletal, pulmonary, renal, gastrointestinal, and vascular systems. Before the identification of the mediastinal mass, the present patient's SS had involved multiple systems and developed cryoglobulin, leukocytoclastic vasculitis, and mononeuritis multiplex. As a well-known risk factor for lymphoma, SS is associated with a risk of NHL 44 times greater than in a normal population [4]. Compared to other systemic autoimmune diseases, SS is associated with the highest risk for NHL development, with a standardized incidence rate of 18.9 (95% CI 9.4–37.9) [13]. Although the precise mechanisms have not been elucidated, chronic stimulation by exoantigen or autoantigen may play an important role in the development of NHL in SS patients, by driving the proliferation of specific B cells and increasing the frequency of their transformation. Additional oncogenic events, such as inactivation of tumor suppression genes and/or activation of protooncogenes, may also be required before clones become malignant and are capable of widespread dissemination and growth [2]. The prevalence of NHL in SS is about 5% and the median time from SS diagnosis to lymphoma diagnosis is 7.5 years [4]. Various subtypes of NHL have been described in SS. MALT lymphoma has been identified as the most common NHL in SS patients [14] and thymic MALT lymphoma has been reported in patients with SS [15]. Compared to MALT lymphoma in general, thymic MALT lymphoma has some distinct features: predilection for East Asians (especially from Japan), strong association with autoimmune diseases especially with SS syndrome, cyst formation, and IgA expression of the tumor cells [15]. Histologically, thymic MALT lymphoma is characterized by centrocyte-like cells and infiltrated Hassall's corpuscles, forming lymphoepithelial lesions [16, 17]. In addition to MALT lymphoma, other B cell lymphomas are also associated with SS. However, THRLBCL is extremely rare in patients with SS. So far, there is only one reported case of THRLBCL (in parotid gland) in SS patients [5].

In the present case, both follicular lymphoid hyperplasia in the thymus and prominent atypical lymphoid hyperplasia in multiple lymph nodes were noted. Reactive lymphoid hyperplasia is a benign nodular lesion, histopathologically characterized by marked proliferation of nonneoplastic, polyclonal lymphocytes forming follicles with an active germinal center [18, 19]. Follicular hyperplasia or atypical lymphoid hyperplasia is not uncommon in SS [20]. Whether the lymphoid hyperplasia in the present patient provided fertile ground for her lymphoma remains a speculation.

THRLBCL is an aggressive lymphoma. Patients often present with an intermediate to high risk International Prognostic Index (IPI) score and the 3-year overall survival is only 46% [1, 21]. The poor outcome is probably related to the advanced stage of disease at diagnosis. The disease is often disseminated at the time of diagnosis because 57% to 82% of patients present with Ann Arbor stage-III and stage-IV disease [21]. However, when patients are well matched according to the IPI and the treatment is adapted to the disease risk, THRLBCL and conventional DLBCL have similar outcomes (response and survival) after chemotherapy. Therefore, after scoring patients according to their IPI, therapy should be similar in THRLBCL and conventional DLBCL patients [21]. The poor results observed in some patients with THRLBCL could be explained by an inappropriate initial therapy rather than a more aggressive clinical pattern [22]. Cases with extension to adjacent organs or thoracic structures (as seen in the present patient), pleural or pericardial effusion, or poor performance status have been associated with unfavorable clinical outcome. On recurrence, the number of atypical cells may increase, resulting in a picture of DLBCL, which indicates a bad outcome [23].

In conclusion, we present the first case of THRLBCL in the thymus. This patient had a long history of SS and lymphoid hyperplasia was also identified in the thymic mass and lymph nodes. In the thymus, the lymphocyte-rich background of THRLBCL can easily mimic lymphocyte predominant thymoma. Recognition of thymic THRLBCL is important for appropriate clinical management. Inappropriate initial therapy may result in worse outcome. The poor results in some of the cases described in literature may have been related to inappropriate initial diagnosis and therapy rather than being due to an inherently more aggressive biological behavior. Delayed diagnosis and inadequate initial therapy may comprise the potential for salvage and long-term survival. Physicians should be careful not to overlook this disease when a patient with a thymic tumor is suffering from autoimmune diseases, especially SS.
